# MoleQCage: Geometric High-Throughput Screening for Molecular Caging Prediction

**DOI:** 10.1021/acs.jcim.4c01419

**Published:** 2024-12-12

**Authors:** Alexander Kravberg, Didier Devaurs, Anastasiia Varava, Lydia E. Kavraki, Danica Kragic

**Affiliations:** † School of Electrical Engineering and Computer Science, KTH Royal Institute of Technology, Stockholm 10044, Sweden; ‡ Department of Computer and Information Sciences, University of Strathclyde, Glasgow G1 1XH, United Kingdom; ¶ Department of Computer Science, Rice University, Houston, Texas 77005, United States

## Abstract

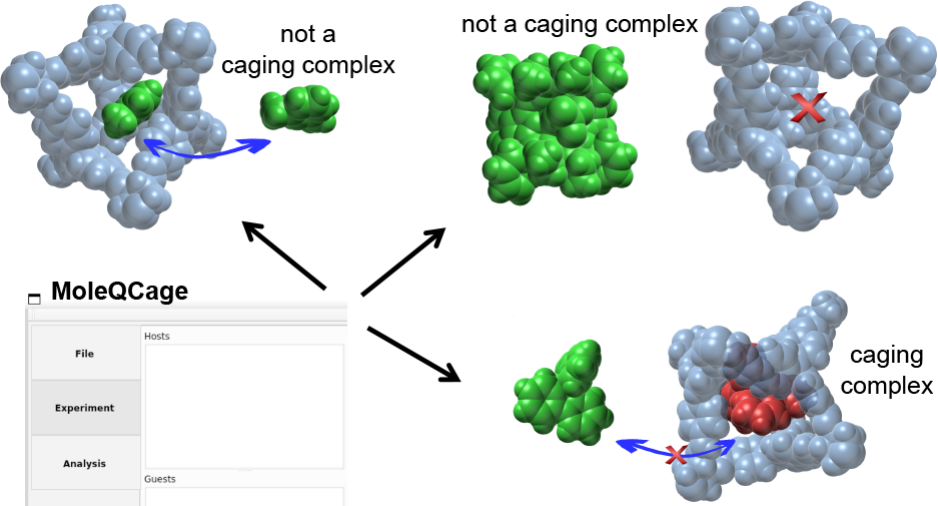

Although being able to determine whether a host molecule can enclose a guest molecule and form a caging complex could benefit numerous chemical and medical applications, the experimental discovery of molecular caging complexes has not yet been achieved at scale. Here, we propose MoleQCage, a simple tool for the high-throughput screening of host and guest candidates based on an efficient robotics-inspired geometric algorithm for molecular caging prediction, providing theoretical guarantees and robustness assessment. MoleQCage is distributed as Linux-based software with a graphical user interface and is available online at https://hub.docker.com/r/dantrigne/moleqcage in the form of a Docker container. Documentation and examples are available as Supporting Information and online at https://hub.docker.com/r/dantrigne/moleqcage.

## Introduction

**Figure 1:**
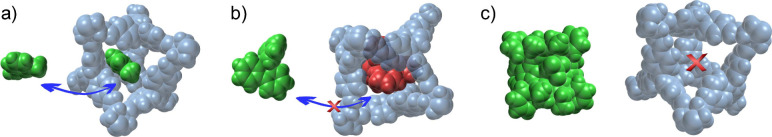
Definition of a molecular caging complex. (a) The guest molecule (in green) can move in and out of the cavity of the host molecule (in blue) and is therefore not caged. (b) The guest fits in the cavity of the host and is either outside (in green) or inside (in red) without the possibility to escape; in this case, the host and guest form a molecular caging complex. (c) The guest cannot fit in the cavity and thus cannot be caged by the host.

In this work, a molecular caging complex is defined as a pair of molecules in which a so-called host (or cage) features an internal cavity that can enclose a so-called guest, preventing its escape (Figure 1). In this kind of supramolecular interaction, we can say that the host cages the guest or, dually, that the guest is caged by the host. In synthetic chemistry, a host molecule is usually created with dynamic covalent bonds, allowing its self-assembly around a guest molecule and its later disassembly in response to a specific stimulus (such as temperature, pH, or light). This paradigm has produced exciting biochemical applications, for example, in targeted drug delivery, virus trapping, or medical imaging.^1−3^ Despite its promises, the use of molecular caging complexes remains challenging, with the discovery or synthesis of host molecules being the main bottleneck.^4^

Strategies for the creation of new molecular caging complexes depend on the application. For example, if a given host is considered for molecular shape sorting, then one has to screen potential guests. In a dual manner, if a particular drug is considered for targeted delivery by a nanoscale carrier, then one has to screen potential host molecules. Unfortunately, current experimental challenges hamper such high-throughput screening efforts and, in turn, make general synthetic approaches very time- and resource-demanding.^5^ This issue clearly highlights the need for computational methods for the high-throughput screening of host and guest candidates prior to experimental validation.

In previous work, we proposed a computationally efficient algorithm to predict if a given pair of molecules are likely to form a caging complex, based solely on geometric considerations.^6^ This algorithm takes two static molecular geometries of arbitrary shape as input; in other words, each molecule is represented by a three-dimensional union of balls of given radii, according to the classical hard-sphere model. Then, as our algorithm is based on a mathematically provable and conservative verification of the caging property, it predicts that a given host–guest pair forms a caging complex only when appropriate theoretical guarantees are met. Note that our caging verification algorithm was initially developed in the field of robotics (for applications to manipulation and path planning), where related concepts of caging were studied. It is important to stress that, as our caging prediction approach is purely geometric, it is different from (yet complementary to) approaches that aim to make caging predictions based on assessing binding affinities.^7^

In this article, we present MoleQCage, a high-throughput screening tool for molecular caging prediction, based on our robotics-inspired caging verification algorithm.^6^ MoleQCage takes as input a set of candidate host molecules and a set of candidate guest molecules. Then, for each pair of host–guest molecules, the underlying verification algorithm determines whether they are likely to form a caging complex, based solely on their geometries, and MoleQCage provides as output a prediction on whether this pair forms a caging complex (+) or not (−). In addition, MoleQCage can consider uncertainties in the definition of molecular geometries and assess the robustness of each caging prediction.

## Caging Verification Algorithm

Given fixed conformations of a host and guest molecules, our algorithm uses an efficient representation of the (six-dimensional) configuration space of the guest to approximate its free space, i.e., the space in which the guest can move within the constraints imposed by the host.^6^ A configuration of the guest molecule refers to its position and orientation in three-dimensional space. If the free space of the guest contains a bounded connected component (i.e., a finite-sized subspace in which every pair of configurations can be connected by a collision-free path), then we can prove that the guest is caged by the host.

In our method, molecular geometries are defined as unions of balls with atomic van der Waals radii, and uncertainties in these geometries are accounted for by varying the balls radii.^6^ This is done by modifying all radii using a given *Δr* value. Varying these radii allows one to assess the robustness of a caging prediction for a given host–guest pair by applying our caging verification algorithm to molecular geometries of slightly different sizes. Indeed, in cases where a host–guest pair might be predicted to form a caging complex based on given molecular geometries, small changes in their sizes might lead to a different prediction. In such cases, either because the guest can now escape or cannot fit the host cavity any more, we say that this host–guest pair forms a “weak” caging complex. In other cases, if the guest is consistently predicted to be caged by the host, we say that the host–guest pair forms a “strong” caging complex; if the guest is consistently predicted to not be caged by the host, we say that the host–guest pair does “not” form a caging complex. Therefore, for each evaluated pair of host–guest molecules, after applying the caging verification algorithm with radii perturbations based on a given *Δr* value (typically ±0.3 Å) in MoleQCage, we can determine whether this pair of molecules (i) does “not” form a caging complex, (ii) forms a “weak” caging complex, or (iii) forms a “strong” caging complex. It is important to insist on the fact that these notions of strong and weak caging have nothing to do with the notions of strong and weak binding affinity.

## Caging Prediction Use Cases

MoleQCage provides users with a flexible graphical user interface (GUI). To define molecular geometries, users can provide as input any file type containing atomic coordinates (such as mol2, pdb, or xyz). MoleQCage can then be applied to several caging prediction tasks.

### Host–Guest Pairs Screening with Robustness Assessment

MoleQCage can be used to screen a large number of guest molecules against a large number of host molecules. As the underlying caging verification is based on a geometric analysis, it is highly efficient and therefore allows for such high-throughput screening. When users provide a set of candidate guests and a set of candidate hosts, MoleQCage runs our molecular caging verification algorithm for all host–guest pairs of molecules, using multiple threads for computational efficiency. Based on the *Δr* value provided by users (or the default value), the robustness of these predictions can be assessed in MoleQCage, so that one can obtain a two-dimensional array with the values “weak”, “strong”, or “not”, for all host–guest pairs.

**Figure 2:**
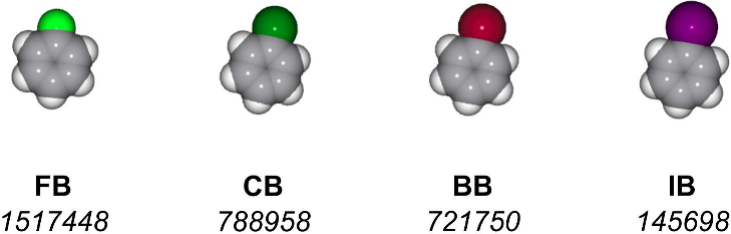
Monohalobenzenes evaluated as potential guests in the host–guest pair screening use case. Each column includes a molecular structure, an abbreviated name, and the identifier of the corresponding CCDC database entry. FB - fluorobenzene, CB - chlorobenzene, BB - bromobenzene, IB - iodobenzene.

**Figure 3:**
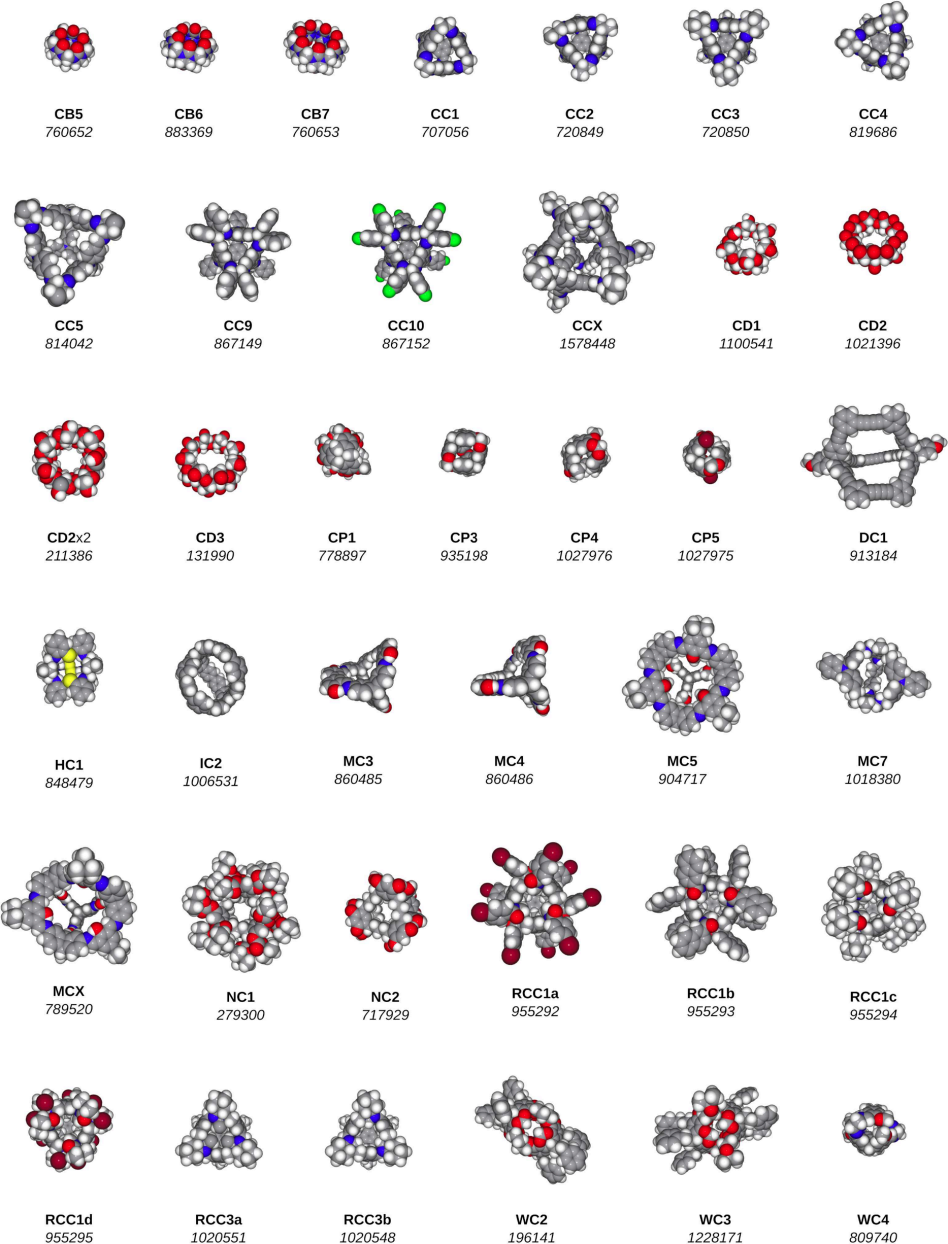
Set of 38 candidate hosts. For each candidate host, we provide a molecular structure, an abbreviated name, and an identifier of the corresponding CCDC database entry.

To illustrate this use case, we consider a set of four candidate guests (Figure 2), which are monohalobenzenes with relatively similar shapes and molecular volumes:^8^ bromobenzene (BB), chlorobenzene (CB), fluorobenzene (FB), and iodobenzene (IB). Note that all crystal structures used in this work were obtained from the Cambridge Crystallographic Data Centre (CCDC) database. In addition, we consider a set of 38 candidate hosts (Figure 3), which are shape-persistent molecules with internal cavities. This list is a modified version of the CDB41 database,^9^ from which duplicates have been removed and to which a few molecules have been added.^10,11^ This experiment is the same as one of those we performed in previous work, but without the duplicate host molecules.^6^

In this scenario, using the default value for *Δr* (i.e., 0.3 Å), MoleQCage allowed us to efficiently screen all 152 host–guest pairs. Results show that 19 host–guest pairs are predicted to be strong caging complexes, 21 host–guest pairs are predicted to be weak caging complexes, and 112 host–guest pairs are predicted not to form caging complexes (Table 1). Among the 19 strong caging complexes, 16 were formed by four hosts (CB6, RCC3b, WC2, and WC3) with four guests. Three other hosts (CC3, NC1 and WC4) are predicted to form strong caging complexes but only with FB (fluorobenzene, the smallest candidate guest), suggesting that their internal cavities are too small to fit larger candidate guests. Among all 38 candidate hosts, WC4 is the only one that is associated with the three possible outcomes, as it is predicted to form a strong caging complex with FB, a weak caging complex with CB and BB, and not a caging complex with IB. As a consequence, WC4 would be a good candidate for the separation of monohalobenzenes.

**Table 1:**
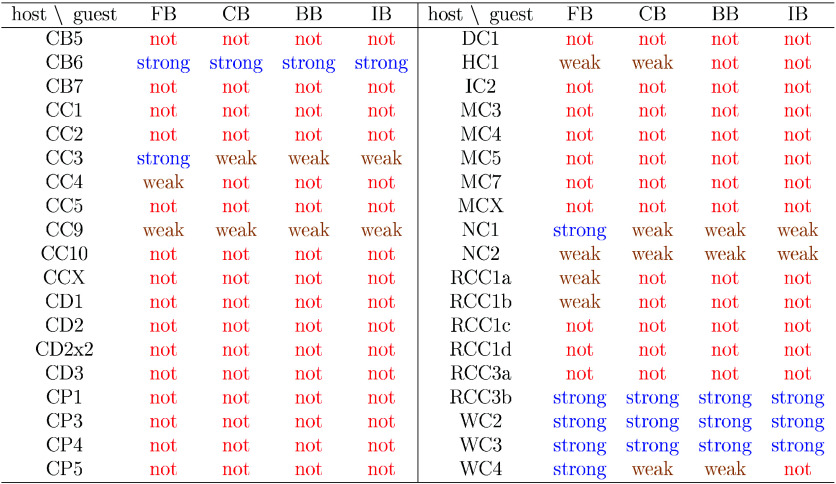
Results Produced by MoleQCage on the Host–Guest Pair Screening Use Case, Which Involved Four Candidate Guests (Figure 2) Listed in the Columns and 38 Candidate Hosts (Figure 3) Listed in the Rows^a^

a For each of the 152 host–guest pairs, the prediction is reported as a **strong** caging complex, a **weak** caging complex, or **not** a caging complex.

### Hosts or Guests Screening with Implicit Molecular Flexibility

As our caging verification algorithm analyzes static conformations of molecules, it does not explicitly account for molecular flexibility. However, MoleQCage allows one to account for implicit molecular flexibility. For that, instead of providing a set of different hosts (or guests), users can provide a set of conformations for a single host (or guest). These conformations can be obtained from structural databases (such as the Protein Data Bank) or via molecular simulations such as molecular dynamics (MD). Therefore, users can screen a set of guest candidates against multiple conformations of a given host molecule or conversely screen a set of host candidates against multiple conformations of a given guest molecule.

**Figure 4:**
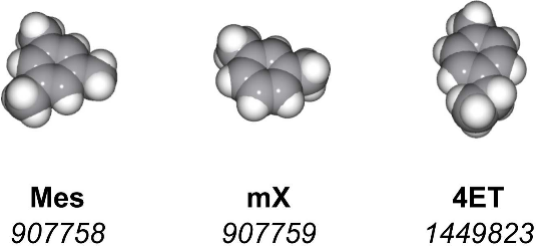
Molecules were evaluated as potential guests in the guest screening use case involving host flexibility. Each column includes a molecular structure, an abbreviated name, and the identifier of the corresponding CCDC database entry. Mes - mesitylene, mX - m-xylene, 4ET - 4-ethyltoluene.

To illustrate this use case, we consider a set of three candidate guests (Figure 4): mesitylene (Mes), *m*-xylene (mX), and 4-ethyltoluene (4ET). As host molecule, we consider CC3 (Figure 3) and use a conformational ensemble containing 515 conformations produced by an MD simulation reported in related work.^12^ These molecules, and the MD simulation of CC3, were already involved in our previous work,^6^ but that previous experiment did not feature a robustness assessment, contrary to what we are reporting here.

**Figure 5:**
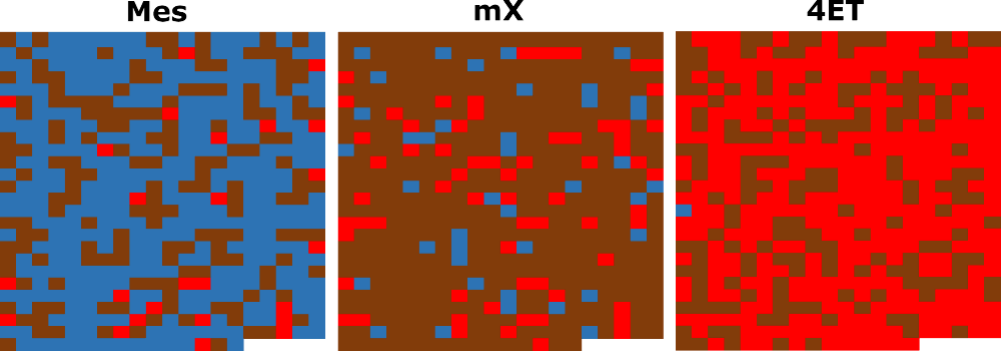
Results produced by MoleQCage on the guest screening with the host flexibility use case. This scenario involved three candidate guests called Mes, mX, and 4ET (Figure 4) and 515 conformations of candidate host CC3. For each host conformation, the prediction is reported as a blue square for a strong caging complex, a brown square for a weak caging complex, or a red square if this is not a caging complex.

In this scenario, using a value of 0.1 Å for *Δr*, MoleQCage allowed us to efficiently screen all 1,545 host–guest pairs. Results show that CC3 forms a strong caging complex with Mes, and forms a weak caging complex with mX, but does not form a caging complex with 4ET (Figure 5). Indeed, 323 out of 515 CC3 conformations (63%) were predicted to form a strong caging complex with Mes; 408 out of 515 CC3 conformations (79%) were predicted to form a weak caging complex with mX; and 340 out of 515 CC3 conformations (66%) were predicted to not form a caging complex with 4ET. This is in agreement with experimental results reported for these host–guest candidates, which showed that 4ET could easily travel through CC3’s windows, that mX could escape CC3′s cavity but not as easily as 4ET, and that Mes was properly caged by CC3.^13^

### Caging Prediction for a Host–Guest Pair with Implicit Molecular Flexibility

Users can restrict their analysis to a single host–guest pair and implicitly consider the flexibility of both molecules by providing a set of host conformations and a set of guest conformations. As in other use cases, MoleQCage will allow users to produce a two-dimensional array containing the values “weak”, “strong”, or “not”, for all conformation pairs. Since the caging verification algorithm is computationally efficient, it is totally realistic to consider screening a large number of host/guest conformations and therefore obtain a caging prediction almost as accurate as if molecular flexibility was explicitly modeled.

**Figure 6:**
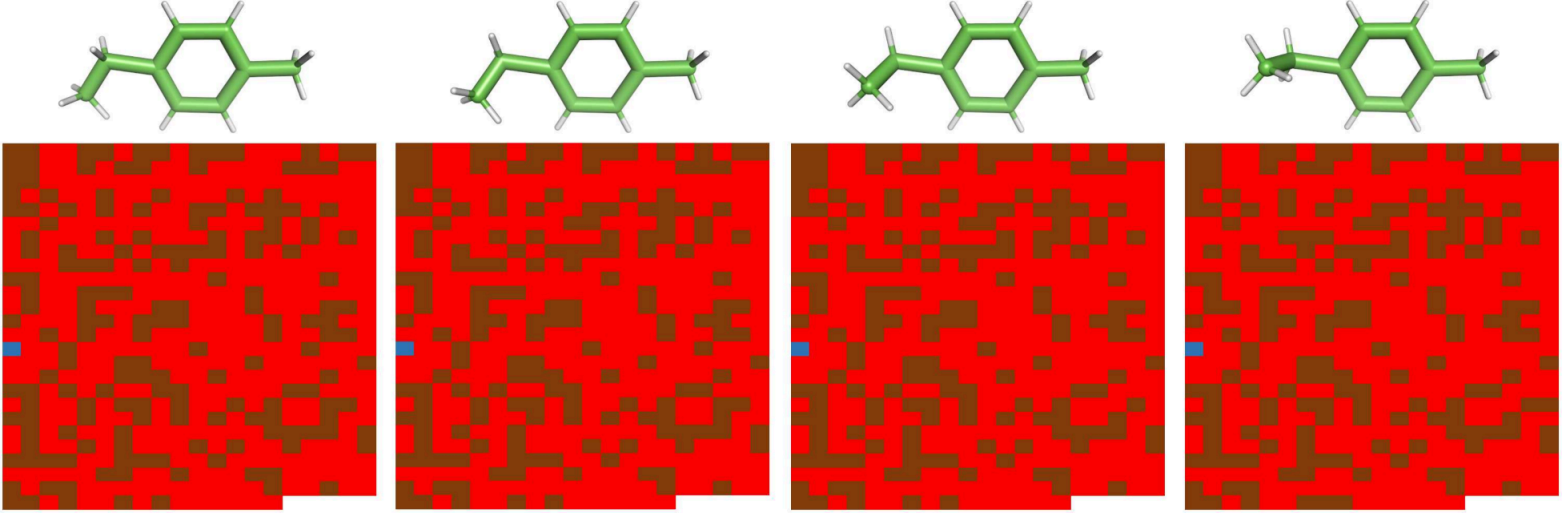
Results produced by MoleQCage on the caging prediction with the molecular flexibility use case. This scenario involved four conformations of the candidate guest 4ET and 515 conformations of the candidate host CC3. For each host conformation, the prediction is reported as a blue square for a strong caging complex, a brown square for a weak caging complex, or a red square if this is not a caging complex.

To illustrate this use case, we evaluate 4ET (Figure 4), using four manually generated conformations, against CC3, using the 515 MD conformations mentioned in the previous section. In this scenario, using a value of 0.1 Å for *Δr*, MoleQCage allowed us to efficiently screen all 2,060 host–guest pairs. Results are very similar to what was obtained without considering the flexibility of 4ET because this small molecule has only one rotatable bond (Figure 6). Therefore, the prediction is still that 4ET and CC3 do not form a caging complex.

## Conclusion

We have presented a computational tool, called MoleQCage, for the efficient screening of molecules to determine whether they can form molecular cages, based solely on geometric considerations. This tool is based on a robotics-inspired algorithm that was presented and evaluated in previous work.^6^ Here, our focus has been on presenting various caging prediction use cases to showcase the versatility and efficiency of MoleQCage. We have shown that MoleQCage can efficiently assess large numbers of pairs of molecules and that it can implicitly account for host and/or guest flexibility, if users provide conformational ensembles for these molecules.

In practice, choosing the right value for *Δr* is not trivial, although we have often noticed that setting *Δr* to 0.3 Å was a good way to produce a relevant robustness assessment. Users should always consider comparing their results to what they obtain with smaller values of *Δr*. For example, the last two use cases mentioned here involve results obtained with *Δr* = 0.1 Å, as this leads to more striking differences among the three candidate guests. Unfortunately, we currently do not have a good way to systematically determine what the ideal value for *Δr* should be for a given experiment.

In solution, the creation of real molecular cages is often driven by supramolecular interactions, such as hydrophobic effects.^7^ Therefore, a clear limitation of our method is that it only considers the geometric shapes of molecules to determine whether they can cage each other. However, our approach can be extended to account for molecular interactions by reformulating it as an *energy-bounded caging problem*, which would be based on the use of an energy field (where a collision could be defined, for example, as *E* ≥ *E*_0_ + *ΔE*), as discussed in our previous work.^6^ We plan to perform this kind of extension in future work.

## Data Availability

MoleQCage is distributed as Linux-based software with a graphical user interface. It is available online free of charge at https://hub.docker.com/r/dantrigne/moleqcage in the form of a Docker container. Documentation and examples are available as Supporting Information and online at https://hub.docker.com/r/dantrigne/moleqcage. Data used to test and validate MoleQCage are provided as Supporting Information.

## Supporting Information Available

Instructions on how to install and use MoleQCage (PDF)
mol2 files of molecules used to test and validate MoleQCage (ZIP)
Rotation grids used to decompose the space of all possible rotations (ZIP)
