# Supplementary material for: MoleQCage: Geometric High-Throughput Screening for Molecular Caging Prediction
Source: J Chem Inf Model. 2024 Dec 12;64(24):9034–9. doi: 10.1021/acs.jcim.4c01419 (PMC11684736; doi:10.1021/acs.jcim.4c01419)
Supplement: Supplementary file 1 — ci4c01419_si_001.pdf [file ci4c01419_si_001.pdf]

Supplementary Materials for
MoleQCage: Geometric high-throughput screening for molecular caging prediction

Alexander Kravberg [1,+], Didier Devaurs [2,*,+], Anastasiia Varava [1], Lydia E. Kavraki [3] and Danica Kragic [1,*]

[1] School of Electrical Engineering and Computer Science, KTH Royal Institute of Technology, Stockholm, Sweden

[2] Department of Computer and Information Sciences, University of Strathclyde, Glasgow, United Kingdom

[3] Department of Computer Science, Rice University, Houston, United States

[*] To whom correspondence should be addressed: didier.devaurs@strath.ac.uk (DD), dani@kth.se (DK)

[+] These authors contributed equally to this work

1 How to install MoleQCage

MoleQCage has been primarily developed to run on any Linux distribution. It can also run on Windows, using WSL (Windows Subsystem for Linux), but performance will be sharply reduced in comparison to Linux. It has not yet been tested on macOS.

MoleQCage is currently released as a Docker container. Instructions on how to install Docker on various platforms are available at the official Docker web site:
`https://docs.docker.com/engine/install`
For example, on Ubuntu, the Docker engine can be installed using the apt repository:
`https://docs.docker.com/engine/install/ubuntu/#install-using-the-repository`

To download the Docker image of MoleQCage, one can run the following command in a terminal:
```
sudo docker pull dantrigne/moleqcage
```
For better performance, one can also download a parallelized version of MoleQCage with the following command, which has to be done only once:
```
sudo docker pull dantrigne/moleqcage:parallel
```

For the Qt-based GUI of MoleQCage to function properly, one has to type the following command in a terminal to create the connection with the X server:
```
xhost +local:docker
```
This has to be done in every new terminal session. Alternatively, this command can be added to the user's .bashrc file and it will be run automatically when the terminal starts.

Typing the following command in the terminal launches the MoleQCage GUI:
```
sudo docker run --rm --network=host --volume="$HOME/.Xauthority:/root/.Xauthority:rw"
 --env DISPLAY=${DISPLAY} -v /tmp/.X11-unix:/tmp/.X11-unix -v <absolute_file_path>:/root
 dantrigne/moleqcage:parallel
```
Here, the `--rm` option ensures that the Docker container is destroyed when the MoleQCage GUI is closed. The <absolute_file_path> is the user's local folder where the files describing the molecules are stored. Inside the container, it will be mounted at `/root`, allowing the user to load these molecules and save results. The other options ensure a proper communication between Docker and the X server.

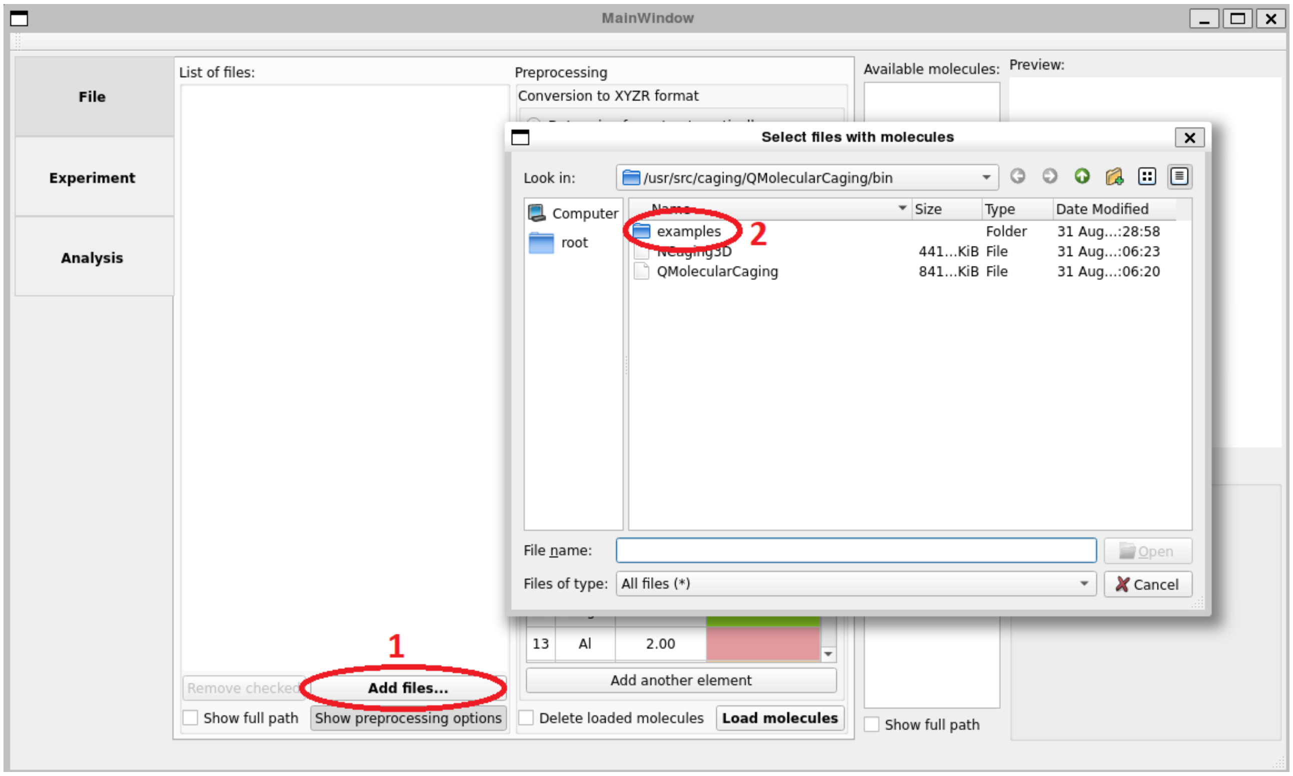

Figure 1: Screenshot of MoleQCage showing how to add files describing molecules to the *File* tab.

2 How to use MoleQCage

MoleQCage opens on the *File* tab (Figure 1). Clicking on the *Add files...* button opens a file navigator at the `/root` directory. If the user did not provide a `/root` directory, the file navigator shows the default location, where the *examples* folder is available. In this folder, one can select the *CC3.mol2* and *mesitylene.mol2* files as examples of a candidate host-guest pair (Figure 2). Clicking on the *Open* button adds these two files to the *List of files* section. Then, clicking on the *Load molecules* button adds these two molecules to the *Available molecules* section.

Opening the *Experiment* tab allows the user to define a set of caging prediction jobs. For example, ticking the box next to CC3 and then clicking on the *Add to Hosts* button adds this molecule to the *Hosts* section (Figure 3). Similarly, ticking the box next to mesitylene and then clicking on the *Add to Guests* button adds this molecule to the *Guests* section. Then, the user has to add a rotation grid by clicking on the *Add grids* button, which opens the file navigator, where the *normal.dat* file is available as the default grid (Figure 4). This grid, as well as all the grids provided as supplementary file, defines the decomposition of SO_3, the space of all possible rotations, in a union of small neighborhoods. The finer the resolution is, the larger the grid file is, and the more computationally expensive the algorithm becomes. The grid referred to as *normal.dat* provides an excellent compromise between accuracy and efficiency.

The user can apply a change in the value of Δr by updating the value of *Epsilon* accordingly in the *Settings* tab (Figure 5), as both parameters are meant to change the values of van der Waals radii. Note that available alternatives include updating the van der Waals radii of the atom types forming the guest molecules when loading them, or uploading XYZR files with modified radii to describe guest molecules while accounting for a change in Δr. Regular users should not try and modify other parameters, as they are made available only for advanced users.

Clicking on the *Add all combinations to the list* button creates a number of computing jobs equal to the number

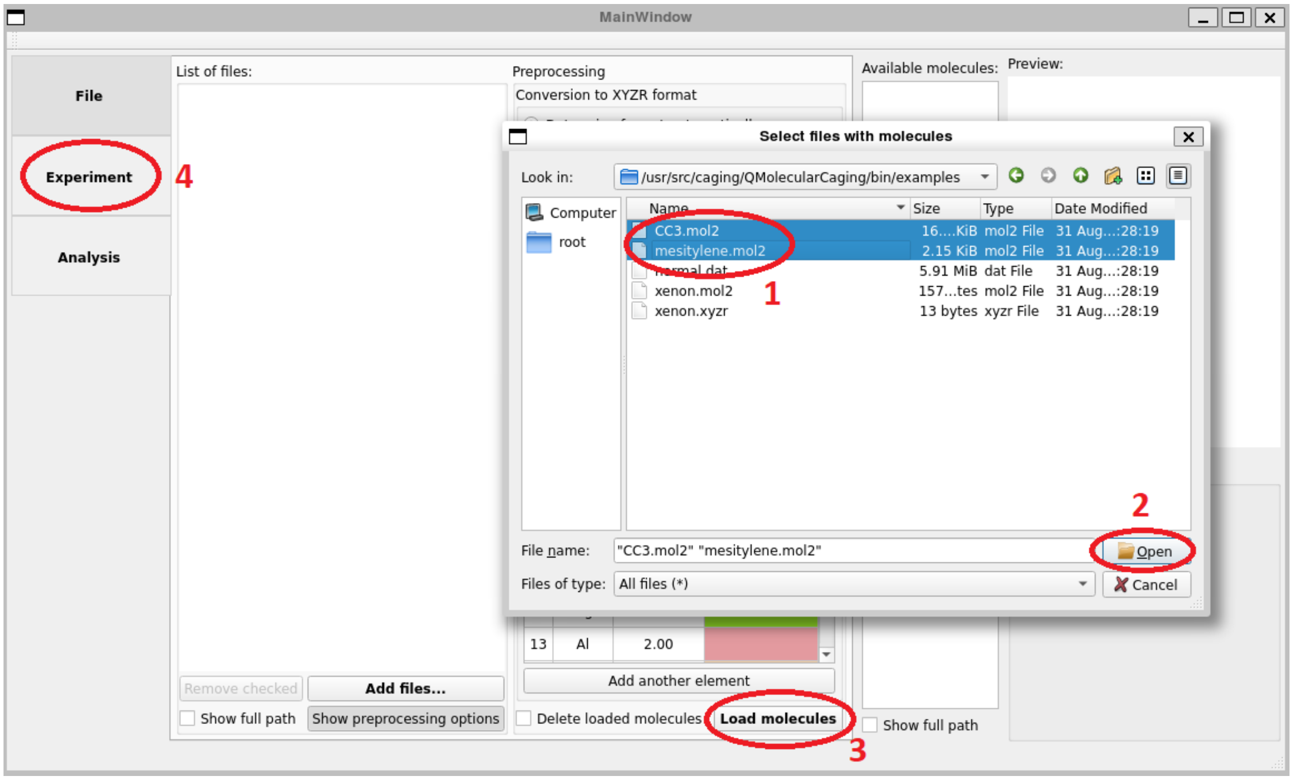

Figure 2: Screenshot of MoleQCage showing two files describing molecules being added to the *File* tab.

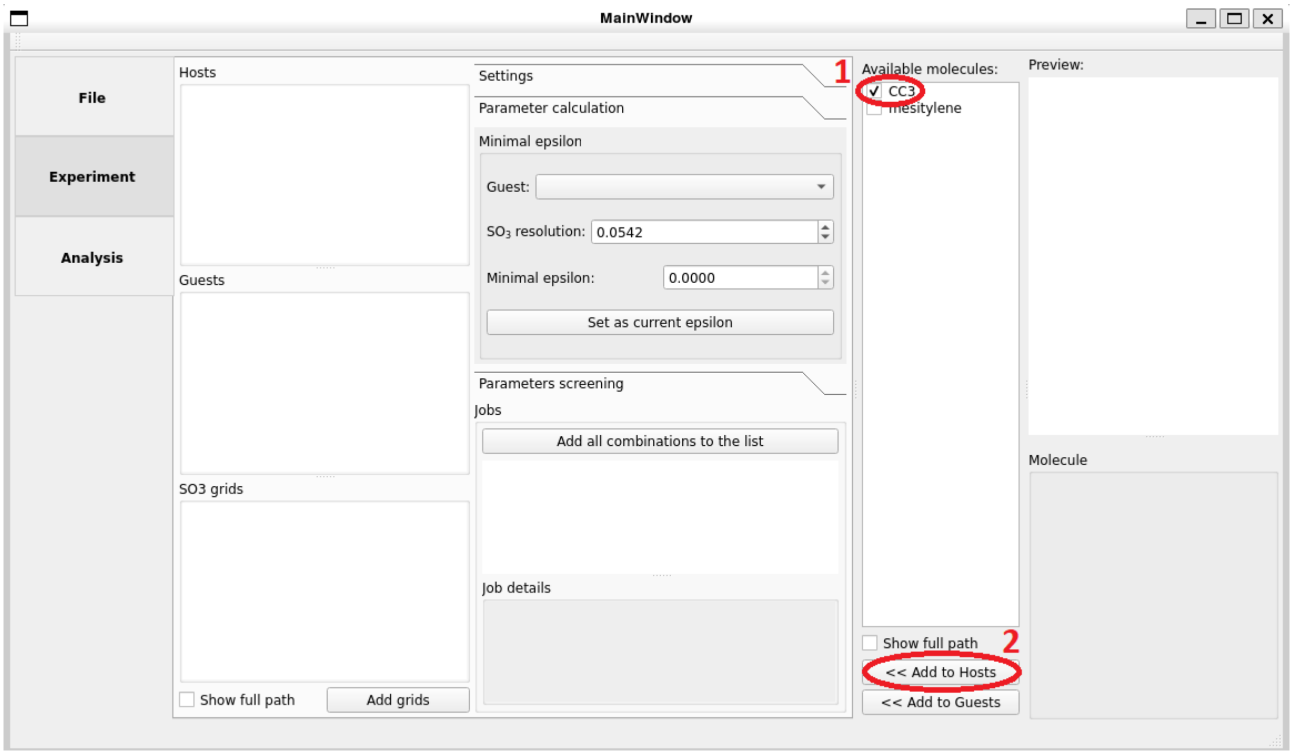

Figure 3: Screenshot of MoleQCage showing how to define caging prediction jobs in the *Experiment* tab.

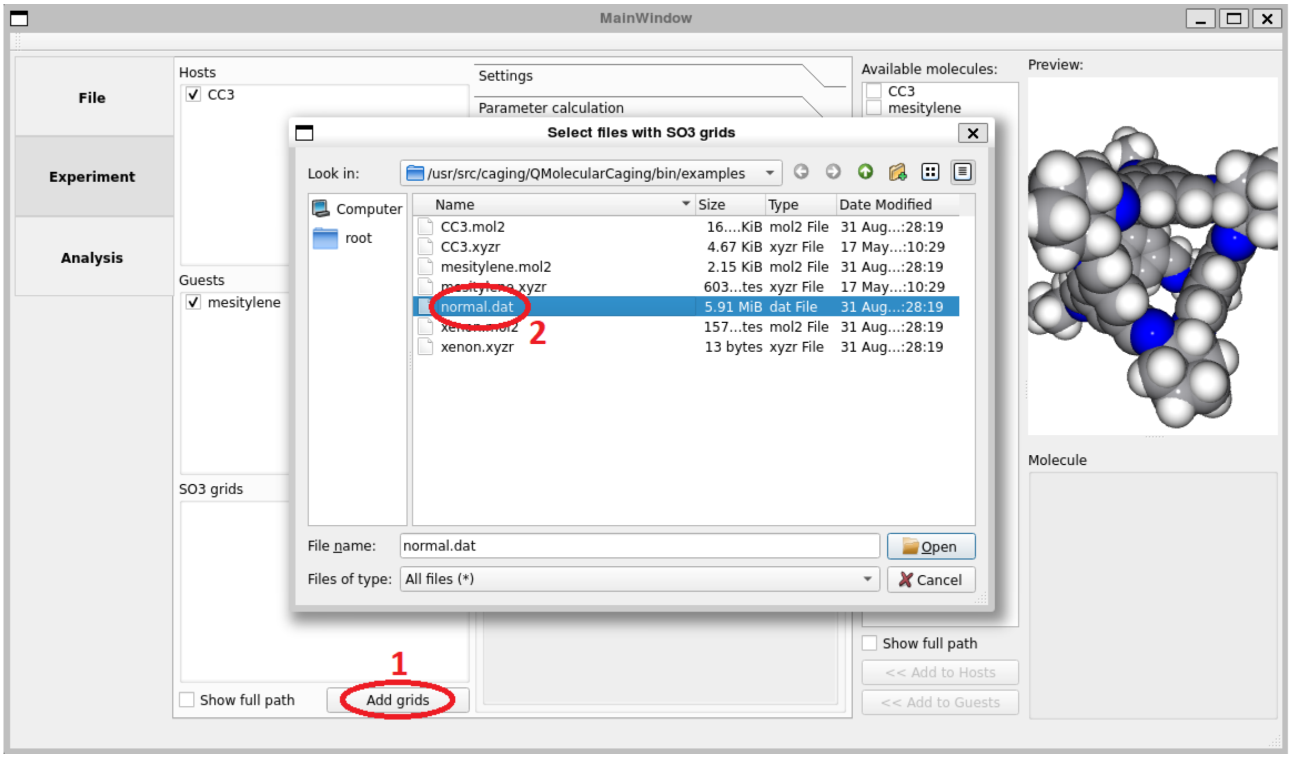

Figure 4: Screenshot of MoleQCage showing how to add the default rotation grid to the *Experiment* tab.

of guests × the number of hosts × the number of grids (Figure 5). Jobs that are running or in queue appear in gold; jobs that have terminated appear in green in the *Jobs* section. When all jobs have terminated, opening the *Analysis* tab allows the user to visualize the caging prediction results by clicking on the *Add jobs to summary* button.

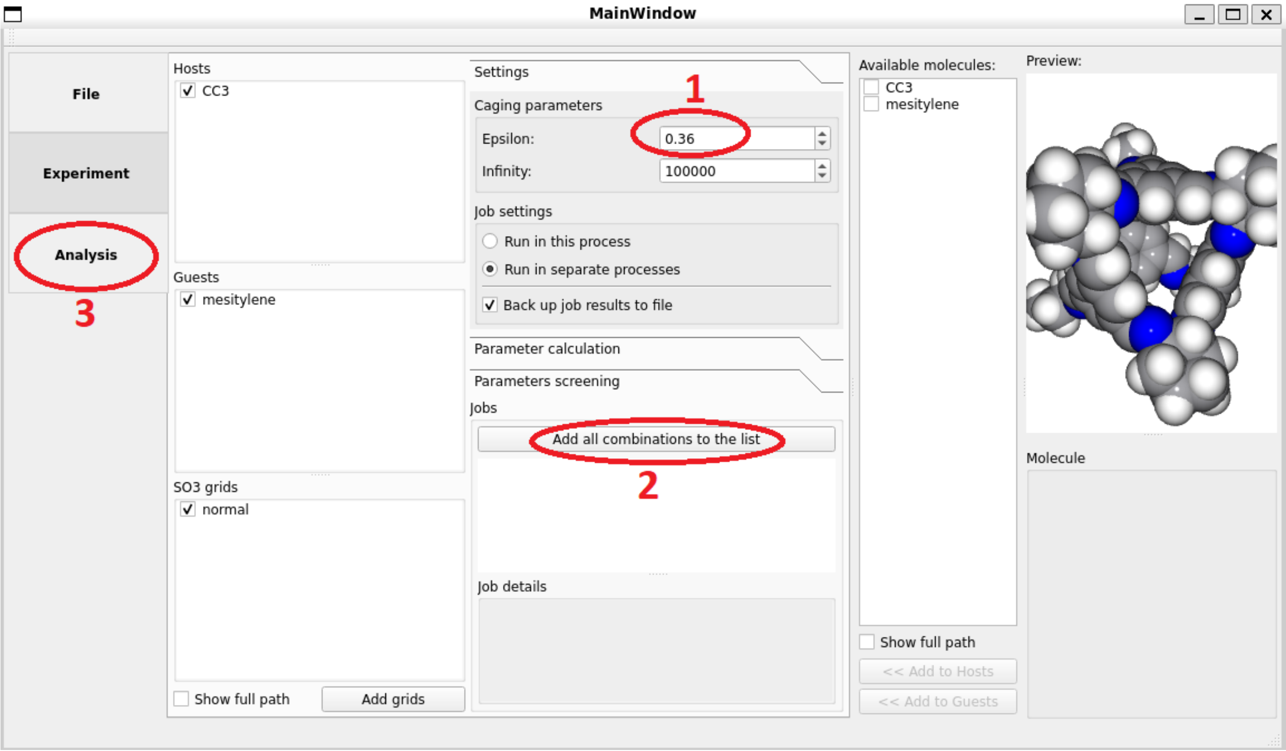

Figure 5: Screenshot of MoleQCage showing how to run caging prediction jobs in the *Experiment* tab.
